# Women's voices and meanings of empowerment for reproductive decisions: a qualitative study in Mozambique

**DOI:** 10.1186/s12978-024-01748-7

**Published:** 2024-02-02

**Authors:** Sofia Castro Lopes, Deborah Constant, Sílvia Fraga, Jane Harries

**Affiliations:** 1 Division of Social and Behavioural Sciences, School of Public Health, University of Cape Town, Cape Town, South Africa; 2 EPIUnit-Institute of Public Health, University of Porto, Porto, Portugal

**Keywords:** Women’s empowerment, Reproductive empowerment, Mozambique, Conceptualization

## Abstract

**Background:**

Women in Mozambique are often disempowered when it comes to making decisions concerning their lives, including their bodies and reproductive options. This study aimed to explore the views of women in Mozambique about key elements of empowerment for reproductive decisions and the meanings they attach to these elements.

**Methods:**

Qualitative in-depth interviews were undertaken with 64 women of reproductive age (18–49 years) in two provinces in Mozambique. Participants were recruited through convenience sampling. Data collection took place between February and March 2020 in Maputo city and Province, and during August 2020 in Nampula Province. A thematic analysis was performed.

**Results:**

Women described crucial elements of how power is exerted for reproductive choices. These choices include the ability to plan the number and timing of pregnancies and the ability either to negotiate with sexual partners by voicing choice and influencing decisions, or to exercise their right to make decisions independently. They considered that women with empowerment had characteristics such as independence, active participation and being free. These characteristics are recognized key enablers for the process of women’s empowerment.

**Conclusions:**

This study’s findings contribute to an expanded conceptualization and operationalization of women’s sexual and reproductive empowerment by unveiling key elements that need to be considered in future research and approaches to women’s empowerment. Furthermore, it gave women the central role and voice in the research of empowerment's conceptualization and measurement where women’s views and meanings are seldom considered.

**Supplementary Information:**

The online version contains supplementary material available at 10.1186/s12978-024-01748-7.

## Plain language summary

Women who are empowered seem to make better health decisions for themselves. Nevertheless, women’s views about and understanding of empowerment are seldom considered in the study of empowerment and its definitions. In this study we explore how women in Mozambique view, understand and experience empowerment, i.e., gaining power and control in the household, and specifically around decision-making processes concerning their reproductive lives. A total of 64 adult women were interviewed in rural and urban areas within two provinces of Mozambique. Through the data analysis, we identified key characteristics of the empowerment process that Mozambican women perceived to be of relevance in their context. Women who have power were perceived as financially and socially independent, free to choose their own pathway, and be active participants in the household decision-making process. In reproductive decisions, women show power through the ability to negotiate with their partner, or by making sole decisions and by planning the number of pregnancies and the size of the family. The elements identified provide important information for improving the definition and the measurement of empowerment in Mozambique, as well as for the support of women in their pathways to empowerment within this context.

**Supplementary Information:**

The online version contains supplementary material available at 10.1186/s12978-024-01748-7.

## Background

Women’s empowerment is a specific target of the Sustainable Development Goals (SDGs 5), making it a development priority in countries and in the international community’s agenda [1]. While evidence suggests that women’s empowerment is generally linked to better health outcomes, findings are inconsistent concerning its contribution to sexual and reproductive outcomes such as fertility and family planning practices [2–5]. These diverse results stem partly from the variety of ways in which empowerment is conceptualized, operationalized and measured in research studies, and partly from the lack of appropriate contextualization of such concepts and measures to the settings in which the studies are conducted [6, 7].

Although Kabeer’s definition of women’s empowerment as ‘a process where women gain power or control to make strategic life choices where this was previously denied’ is widely used [4, 8], there is still no consensus about what empowerment represents and how it should be operationalized and measured. This is demonstrated by the considerable diversity in the terminology used to describe empowerment, and the extent to which terms are used interchangeably. For example, the terms ‘autonomy’, ‘agency’, ‘women’s status’, have been used interchangeably to describe and measure women’s empowerment [4]. Further, many empirical studies do not use theoretically grounded models to guide and refine their conceptualization and definitions of empowerment. These problems have slowed the development of evidence on the association between women’s empowerment and health and reproductive outcomes [4, 6].

In Mozambique, gender inequality and unbalanced gender power relations are considered important determinants of women’s health [9, 10]. Rooted in a patriarchal society, with strong traditions an social and gender norms, women in Mozambique are often not empowered to make decisions concerning their lives, including their bodies and reproductive options [10]. This has resulted in slow progress in the use of modern contraceptive use (from 25.7% in 2015 to 36.4% in 2020) [11, 12] and in reduction of the total fertility rate (from 6.1 in 2015 to 4.6 in 2022) [11, 13]. Women are among the most vulnerable populations in Mozambique, increasing the chances of disempowerment. For example, girls and young women in Mozambique are less likely to complete school than boys and young men [14]; women have less access to paid employment [15], have higher risk of HIV [16], being expose to violence [11] and being undernourished [17]. Therefore, promoting women’s empowerment in Mozambique could accelerate advancements in women’s health while also tackling gender inequality.

Women’s empowerment is intrinsically linked to the specificities of the context in which it is measured [18]. While the core ideas of women’s empowerment are universal, evidence suggests that beliefs, attitudes and behaviors that reflect empowerment in one context might not indicate empowerment in another [19]. An example is the use of empowerment measures primarily developed for Asian countries, such as women’s social mobility. Some of these turned out to be irrelevant to the African context due to social and cultural differences between the two regions [4, 20]. Although standardized measures theoretically allow for comparison between countries, the validity and interpretations of findings should be questioned if no cultural and social understanding exists to support interpretation [4, 6], context-specific indicators might thus be required and of value. In addition, women’s perception and understanding of empowerment, shaped by their values and cultural background, most likely varies across settings [4]. However, this is seldom considered when deciding how to measure or conceptualize empowerment [21].

Incorporating women’s views and meanings—i.e. how they understand and describe empowerment—is key to uncovering underlying aspects of decision-making, access to choice and freedom to choose the options they perceive as best, power structures, and gender dynamics [22]. Although empowerment is about women, their views, meanings and lived experiences are often not considered in the conceptualization and operationalization of the term. The primary goal of this study was to explore the views of women in Mozambique about key elements of empowerment, the intricate meanings they attach to these elements, and which elements they identify as the most relevant to their lives. Those elements might contribute not only to the validation of the way that women’s empowerment is currently conceptualized and operationalized, particularly within the fertility and family planning field, but also support uncovering important insights that can further research on women’s empowerment. By exploring women’s voices, this study also aimed to contribute to inform women-centered programmatic strategies, more adjusted and responsive to women’s needs in Mozambique. This study was part of a larger mixed methods research study that aimed to describe how women’s empowerment influences the reproductive choices of women of reproductive age in Mozambique [23–25].

## Conceptual model

**Fig. 1:**
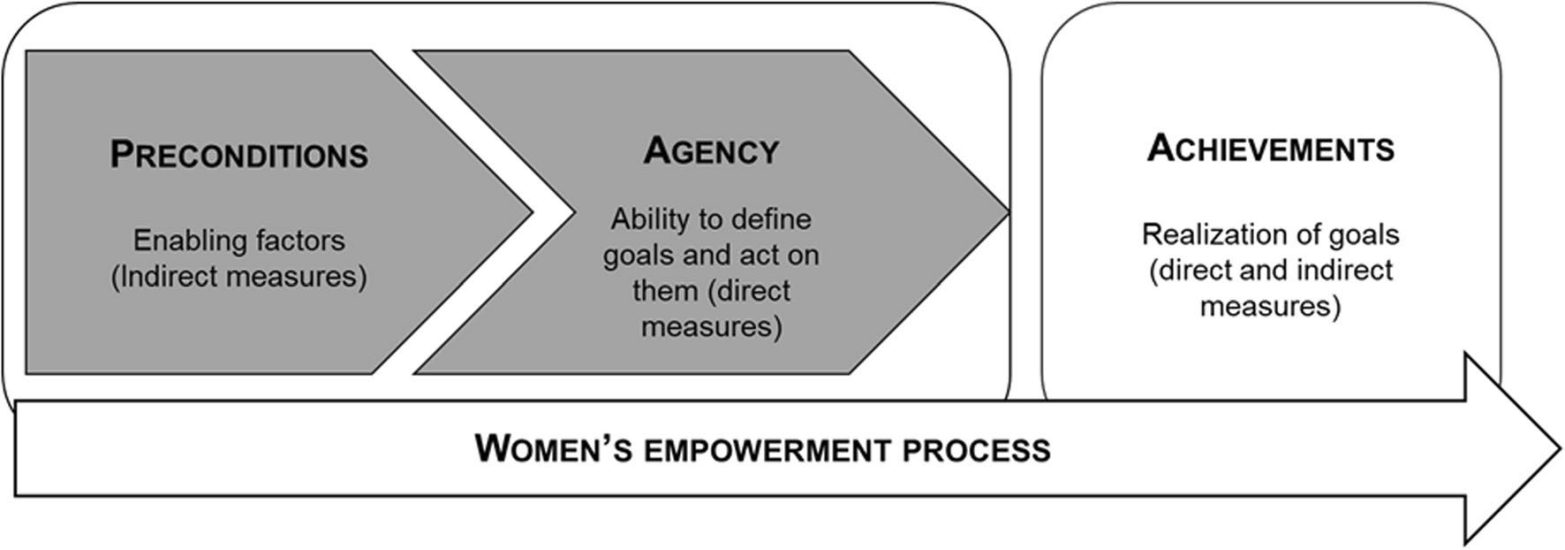
The conceptual model of women’s empowerment process (adapted from [8])

In this study women’s empowerment was defined as *Having the power to control and freely decide over one’s life and body in order to achieve valued or best perceived outcomes* [8, 26]. This definition is based on Kabeer’s foundational work (Fig. 1) on the conceptualization of women’s empowerment [4, 8]. In this model, empowerment results from two essential components—preconditions or resources and agency. Briefly, preconditions or resources are enablers of the empowerment process but do not confer power or control or result in the exercise of choice per se. Rather, they create the conditions in which women’s ability to decide and act are enhanced. Types of resources include material (for example, income), social and human resources (for example, education) [8]. Agency is the ability to define goals, make independent decisions and act upon them [8] guided by meaning, motivation and purpose and deeply based on women’s sense of agency or power-within. Achievements refer to the realization of the set goals [4, 8], such as the decision about childbearing or the use of contraceptives.

In addition to Kabeer’s work, the definition of empowerment used in this study also considers Amarthya Sen capability model on inequalities and recently adapted to the study of empowerment and health [6, 26, 27]. Briefly, Sen’s capability theory says “The focus here is on the freedom that a person actually has to do this or be that – things that he or she may value doing or being according to the extent of freedom people have to promote or achieve functionings they value” [26]. This is based on two essential components: (1) freedom to choose what to be and what to do and (2) valuable functionings (the ‘beings’ or ‘doings’ that a person can choose to achieve).

## Methods

### Study design and setting

A qualitative study was conducted through interviews of Mozambican women to explore their experiences, views and meanings of empowerment in relation to fertility and family planning practices. The methods of this study was previously described elsewhere [25]. Briefly, in-depth interviews were conducted with women of reproductive age (18–49 years) living in the provinces of Nampula in the north, Maputo in the south, and in Maputo city in Mozambique. The selection of these locations was guided by disparities in contraceptive utilization and levels of women's empowerment highlighted in the 2015 Demographic and Health Survey (DHS) [11]. The DHS measures of women’s empowerment include a set of indicators about who usually makes decisions in the household (e.g. health care, purchases and visiting friends or family), if a woman can ask for the use of condoms or refuse sex, and if beating is justified in some situations (e.g. going out without telling the husband, neglects the children, argues with husband, refuses sex or burns the food).

With support from the Provincial Health Directorates (DPS—Direcção Provincial de Saúde) and the Health Directorate of the City of Maputo, the research team sampled five health centers, three urban and two rural. This selection was based on the availability of varied contraceptive methods, the diversity of the served population, and logistical accessibility.

The data collection team comprised a lead researcher (SCL) and a research assistant from Mozambique who provided translation from local languages to Portuguese.

### Study participants and recruitment process

The study sought participation from women of reproductive age (18 to 49 years), not pregnant (confirmed verbally), who attended one of the selected health facilities or lived in the communities served by these facilities. Pregnant women were not included in the study as empowerment levels and experiences might change during pregnancy. Also, pregnancy might influence how women perceive decision-making around fertility and family planning practices. Health facilities were the place of recruitment of participants as it was the most feasible approach given the financial, time and human resources available for the implementation of the study. It was an effective way of reaching women and having a private space for an interview addressing sensitive and intimate aspects of life.

At health centers, the lead researcher approached potential participants individually and in groups in the waiting room. A succinct explanation of the study's purpose and the nature of involvement was provided. Interested women were then taken to a private room within the healthcare facility for a detailed overview of the research. Their eligibility was confirmed, and informed consent was obtained. For participants in the community settings of both provinces, a convenience sampling approach was employed, aided by local community leaders. These leaders identified women of reproductive age who rarely accessed healthcare facilities, who were less likely to use contraceptives and were available for in-depth interviews on predetermined dates. Due to the reduced influence of community leaders in Maputo city, a snowball sampling technique was applied in this setting. Upon agreement, the interviews took place either in the participant's home or a public space.

### Data collection

The research question ‘What views and meanings of empowerment do women of reproductive age identify in their lives?’, together with evidence from published literature, guided the development of a semi-structured interview guide (Additional file 1) [4, 28, 29]. The interviews employed a life timeline technique [30] to elicit participants' empowerment experiences within their reproductive journeys. This approach looked at significant life events, decisions, power dynamics within households, and interactions with healthcare services as they pertained to fertility intentions and family planning. The latter part of the interviews focused on participants' views on gender roles and power dynamics within their communities and Mozambican society more broadly.

Prior to the commencement of data collection, the research assistant underwent training on conducting the interview and obtaining informed consent. The interview guide was tested through pilot interviews conducted in selected urban and rural settings in both provinces. Feedback from these sessions led to improvements in language and terminology to enhance clarity. Regular meetings between the research team were undertaken to prevent or minimize the risk of interviewer bias.

Data collection took place between February and March 2020 in Maputo city and province. Due to the COVID-19 pandemic, data collection in Nampula province had to be postponed to August 2020 when all safety measures could be put in place. Interviews were conducted in Portuguese, the official language of Mozambique. Translation support to local languages was provided when necessary by the research assistant. The interviews had an average duration of 45 min and were audio-recorded.

### Data analysis

The qualitative software package NVivo 12 was used to sort and manage the data [31]. A thematic analysis was conducted using both inductive and deductive approaches [32]. The lead researcher (SCL), a native Portuguese speaker, coded all transcripts sentence by sentence, identifying themes related to reproductive empowerment as perceived by the women. A coding framework was developed to support the organization of the codes that emerged from the analysis under each topic. To ensure rigor and data quality, a triangulation strategy was employed, with the first and last authors collaborating in the coding framework validation.

To prevent loss of meaning and increase the accuracy of the interpretation of the findings, all interviews were transcribed verbatim and analyzed in Portuguese. The translation of illustrative quotes and passages from Portuguese into English was done later in the analysis process.

## Results

In total, 64 women participated in the study: 39 from Maputo city and province (21 from health centers and 18 from the community) and 25 from Nampula (19 from health centers and 6 from the community). Of these, 41 women lived in urban areas, and 23 in rural areas. Participants’ characteristics are described in detail in the supplementary material (Additional file 2). Overall, women from Maputo were slightly older, more educated, and more likely to be single when compared to women from Nampula. In Maputo, 46% of the participants reported being employed, compared with only 12% of women in Nampula. Most of the employed women lived in urban areas in both provinces. All participants from Maputo had used modern contraceptive methods at some point in their lives. In both settings, urban women were more likely than rural women to use contraception. Injectables and oral contraceptives were the preferred methods by participants in Maputo, while in Nampula injections were the most used method. On average, participants living in urban areas of Maputo had their first pregnancy three years later than women in the Maputo rural areas, and they also had fewer children. Such differences were not found between rural and urban areas in Nampula.

From the thematic analysis, two major themes reflecting women’s views and meanings around empowerment were identified: Women’s characteristics associated with empowerment; and women’s actions/manifestations of power.

### Women’s characteristics for empowerment

Under women’s characteristics for empowerment, three sub themes emerged expressing women’s perceptions of what a woman is or looks like when she is generally empowered within the household. The characteristics included being independent, an active participant in decision-making, and being free and experiencing freedom. Such characteristics can also be considered as essential preconditions or determinants for women’s empowerment.

#### Being independent

Overall, women with empowerment were perceived as being independent, particularly in relation to decision-making within the household. These women were characterized as having some level of financial and social autonomy from their husbands, partners, or other family members.

‘I am not very dependent on him. I am also independent financially, so some of the things I just decide on my own - "I am going to do this”(...).’ (Maputo city, 32, married, 2 children)

‘There have been some changes. Women before were dependent on their husbands. Nowadays, women are independent to work and have their own businesses.’ (Nampula, urban area, 23, single, 1 child)

The participants also identified key features of women they perceived as financially and socially independent and with power for decision-making within the household. These included having a job outside the house, and earning an income, as illustrated by these quotes.

‘If a woman has a job, she can make decisions. If a woman brings money to the household, she can make decisions.’ (Nampula, rural area, 23, married, 2 children)

‘(Work) has a very big impact because here the idea is for women to be submissive and wait for the men to support her and all the expenses, so that is why finding a job was so important for me, I felt autonomous.’ (Maputo city, 37, single, no children)

#### Being an active participant in decision-making

Women with empowerment were also described as active participants in different spheres of their lives. Women’s participation was related to having a voice and being heard by their families and communities:

‘We don't accept that men step on us anymore, we don't accept that. Now we are able to speak.’ (Maputo, urban area, 44, married, 3 children)

‘Women must do family planning. It is very good for them. And they must demand what they want and don’t let themselves just die having children. We must set up and demand the life we want.’ (Maputo, rural area, 33, divorced, 3 children)

#### Being free and experience freedom

Being or feeling free was another characteristic identified and valued by women in relation to decision-making processes. Freedom was related to women’s understanding of the right to choose, i.e. the right to self-determination but also the idea of being free from threats. This characteristic was often associated with the experience of wellbeing and satisfaction in freely choosing their own pathways. Such pathways were often different from societal gender expectations around marriage and motherhood as described by these study participants from Maputo:

‘A woman can decide not to have a husband and be very clear about the life she wants to live. What does that mean? It means that she can decide not to have a husband and have a different focus in her life, either studies or a job, and she feels satisfied about it. (…) Today the society we live in gives us the freedom to choose.’ (Maputo city, 34, married, 2 children)

‘I believe she is living in a way that is very good for her. (…) I think she is exercising her freedom. She is doing so well. I never seen anyone being so clear about not wanting to have children, just wanting to work and travel.’ (Maputo city, 29, single, no children)

'I believe women should be free….to do what you want to do. And be free to do it, to not be trapped like “Oh I cannot do this or that because my husband does not allow it.” No.' (Maputo, urban area, 47, 6 children)

### Women’s actions and manifestations of power

Participants identified different ways in which women could manifest power directly or indirectly in relation to their reproductive lives, namely through planning childbearing and family size, negotiation with partner and sole decision-making regarding family planning. These dimensions are linked to manifestations of agency by women, a key component of the empowerment process.

#### Planning childbearing and family size

Despite the value given to motherhood and children being considered by some women as the family’s ‘wealth’, many also valued the ability to plan the number and the timing of pregnancies. Women viewed planning as having options or alternatives in life. The following participant explained how the concept of planning, and family planning in particular, changed her perspective of childbearing:

‘(Family planning) helps you to be healthy and to make your own (life) plan while you are still free. If you have a baby, you cannot plan anymore, life gets complicated. You would like to attend school, but you cannot because you have to look after that baby. So that baby must be planned. If I had done that, I think I would have suffered less but it was before there was information…we did not know much then.’ (Maputo city, 42, married, 3 children)

Different reasons lead women to plan their childbearing. While some women prioritized their own health, for many there was a concern with the children’s future, including their well-being, healthy growth and access to school. This participant explains her motives for not wanting more than 3 children:

‘Your child must be well taken care of. He or she cannot just be uncared for. He needs clothes, he needs to be clean, the house must have good conditions. But if you cannot manage this then everything gets difficult. One can support 3 children, but 4 or 5 is too much. It is hard! It is difficult to have children. Even these three I have, give me stress. They go to school, and they need this, and they need that… So, it is not worth to have more.’ (Maputo, rural area, 31, married, 3 children)

For some participants, planning when to have children and how many to have, was also shaped by their own aspirations and goals:

‘I don't want to have more children now because I am studying. My husband is the one taking care (paying) for my school things. I want to finish high school and then apply for the nursing school. (…) When I have a job, I want to have a bank account to keep my money. I will buy myself hair every month, I will buy things for my daughter, and we will go out for ice-cream.’ (Maputo, urban area, 20, married,1 child)

#### Partner negotiation

Negotiation involved the ability to voice or communicate one’s own wishes or opinions. These processes were often described as ‘sitting down’ or ‘having a conversation’ with their partners. A woman from Maputo described her experience:

‘It is a conversation. It has to be over conversation. And I thank God that there is openness for that, for conversation. So, it is a talk. As soon as I left the hospital and I went home, he asked: "Are you well? When are you going to start family planning?".’ (Maputo city, 34, married, 2 children)

Women’s experiences shed light on some nuances of the negotiation process. Certain aspects of communication were valued, such as the ability to voice and present one’s ideas and the importance of listening to one another. An important element of this is to know what one wants—to have made an ‘internal’ decision—and to be able to present it. A woman from Maputo describes how she experienced this process in relation to family planning:

‘We have a conversation, right, and each of us presents their points of view. So, if I want him to understand my views and my thinking, I need to be able to speak about my reasoning…it is hard to convince a man. It is hard but I need to be able to make him understand my reasons and my decisions. For example, I am going to tell you about my decision about family planning. There are several methods and for him (husband) I should take the injection. I said no. I know my body. Contraceptives react differently in each body, so I had to convince him.’ (Maputo city, 34, married, 2 children)

A woman from rural Maputo describes the importance of listening in terms of reaching consensus, and balancing power in decision-making, which to a certain extent denotes understanding of an entitlement to express and defend one’s own ideas and decisions:

‘Well, I think one has no more power than the other (husband and wife). They must find consensus. And for that they must listen to each other. One cannot just decide without listening to the other.’ (Maputo, rural area, 32, divorced, 3 children)

However, for some women, even if they are able to engage in negotiation and voice their reproductive desires, in the event of a disagreement men have the last word, as described by this participant from Maputo:

‘We should try to reach agreement but if it’s not possible then the woman must follow the man’s indications.’ (Maputo, rural area, 25, married, 3 children)

Furthermore, for some women, especially those from Nampula, the use of words about negotiation was often connected to getting the partner’s permission or acceptance. This is also suggestive that in case of conflicting opinions and desires, the will of the woman might be overpowered. This was noted in the following comment:

‘If you are married you must ask for permission from your husband. But if you are single you can decide about your life. But if I have a partner, every step that I take I must communicate with him. "Look, I want to do family planning"... Family planning is a matter of two people. When I am doing it, he is also doing it. So it is about two people.’ (Maputo, urban area, 33, married, 3 children)

On the other hand, some women stated that their decision about reproductive matters prevails when agreement is not reached in the negotiation process with their partner, even if that necessitates covert action, as observed by this woman from Nampula:

‘Some women tell their partners that they want to stop (having children), as they already have 3 or 4 (children) but if the husband does not accept that, so they do it (use contraceptives) behind their backs.’ (Nampula, urban area, 40, married, 3 children)

#### Sole reproductive decisions

Participants reporting sole decision-making for fertility and family planning believed that such matters only concerned women, because it is related to their own bodies and their own health, they had other aspirations in life, they wanted to provide to their children, or they wanted to find the right partner:

‘I don’t want to have children to leave them to be raised with my parents or with my grandmother. I want to have them at the right moment, with the right person, so I can raise them and I am able to provide for them.’ (Maputo city, 29, single, no children)

For some married women, this was also related to the partner’s lack of interest in family planning, as this woman from Maputo described:

‘Many times, we women, need to decide for ourselves because men don't even care. If you prevent it or not (pregnancy) it is your problem. I think most women take care of themselves (without asking the husband).’ (Maputo, urban area, 44, married, 3 children)

A participant from Nampula highlighted the importance of having access to information about family planning and how that seems to result in feelings of entitlement to make her own decision without the need for her partner’s involvement or to protect herself from threat:

‘I used to listen to some talks here at the health center. After one of those talks, I started thinking… “my partner is aggressive and when he wants (sex) he wants it.” So I decided that I should protect myself. On that day I asked for the pill.’ (Nampula, urban area, 25, married, 3 children)

Some women opted for covert decision-making on the use of contraceptives. This was related to possible opposition or nonacceptance from the partners hindering women’s ability to control their reproductive choices. This noted in the words of this participant from Nampula:

‘So I started considering the consequences for myself: I could have diseases, I could get an unwanted pregnancy. So I just decided to use contraceptives and don't tell him about it. I think he wouldn't accept it because he wants to have children.’ (Nampula, urban area, 20, single, no children)

## Discussion

This study described views of empowerment, highlighting elements (and their meanings) that Mozambican women perceived as the most relevant in their context. These findings align with and complement the qualitative results from the larger research study, describing the facilitators and barriers to women’s empowerment for reproductive decisions in Mozambique [25]. Women with empowerment were perceived as financially and socially independent; as active participants in life by expressing their opinions; and free to choose their own pathways. These characteristics have been identified in the literature as essential preconditions and/or determinants for empowerment [25, 28, 33]. Manifestations of power, also described in the literature as dimensions of women agency [21, 33], for reproductive decision-making included women’s ability to plan their family size; negotiation with sexual partners and reaching consensus or obtaining permission and/or covert action; and sole decision-making. These were fueled by an understanding of their rights and entitlement to decide over their own bodies (bodily autonomy), access to information, or a lack of male engagement.

Some of the identified elements such as negotiation or sole decision-making for reproductive issues have been considered in the literature and incorporated in conceptual models of women’s empowerment [21, 33]. However, the nuances captured in women’s perceptions add layers of understanding to each of these elements which can contribute to refining the conceptualization and measurement of empowerment. Other elements such as the ability to plan the number and timing of pregnancies or the ability to voice choices and wishes, add new dimensions and specificity to women’s reproductive decision-making processes that can help further unpack and understand empowerment, especially in relation to sexual and reproductive health.

The findings of this study emphasize the relevance of a woman’s ability to voice choice and negotiate in her sexual and reproductive decision-making processes [25, 34, 35] These findings align with a recent conceptual framework for Women’s and girls’ empowerment in sexual and reproductive health [21]. In this framework, negotiation skills, decision-making abilities and self-efficacy are key elements of the exercise of choice. The exercise of choice expresses women’s ability to act on their sexual and reproductive preferences, i.e. women’s agency [21]. Although voicing a choice and participating in negotiation are intrinsically connected, the ability to voice a choice does not necessarily equate to the ability to negotiate or influence the decision-making process. The two concepts should therefore be considered independently. The insights provided by this study into key elements of the process of decision-making for reproductive choices can support the refinement of the measurement of agency. Agency is one of the most challenging components—and perhaps the most poorly assessed component—of empowerment in the literature, because direct measures (of action) are generally difficult to operationalize [4].

While many women perceive negotiation as a fundamental process for women’s sexual and reproductive decision-making, it was in some instances connected to the need for a partner’s ‘agreement’ or ‘permission’ and, in case of disagreement, the partner’s opinion often prevailed. This finding highlights the importance of comprehending the negotiation process related to reproductive issues—and the resulting empowerment—by disentangling whether it means a truly mutual decision or rather a partner’s decision. A study by Peterman et al. (2015) found a small correlation between indices that include sole decision-making only and indices that include sole or joint decision-making in relation to women’s agency, highlighting the need for a better understanding of the process of joint decision-making, and whether it allows the woman to act on her choice [36]. For example, assessing if women desire sole control over decision-making or would rather decide jointly with a partner can provide clarity to quantitative findings [35].

Some women consider themselves to be the ultimate decision-makers for reproductive decisions. While some women expressed their entitlement to decide about their reproductive lives because it was a matter that concerned them and their bodies, other women opted for covert sole decision-making, such as the use of contraception without the knowledge of their partner. Covert use of contraceptives was also found among women who negotiate reproductive decisions with their partners, when facing opposition. It is crucial to differentiate between individual or supposedly 'joint' decision-making regarding reproduction and concealed decision-making, a phenomenon prevalent in Mozambique and other sub-Saharan African countries [37]. While covert decision-making might be seen as a form of empowerment, it might not completely align with empowerment’s comprehensive definition and prerequisites. One potential approach could involve investigating whether decisions are made without any influence of coercion, alongside examining women’s perceptions of their entitlement, i.e. the focus would be on whether women believe they have autonomy to make their own choices if they so desire [34, 38].

Many women recognized the ability to plan the number and timing of pregnancies as a manifestation of power or a way of empowerment. This not only gave women a sense of control, but also created a sense of alternatives or options in their lives that could apply beyond childbearing. In the Karp et al. framework, planning ability could be considered within the dimension of understanding the existence of choice, a necessary step for the agency to follow and the overall process of empowerment [21]. In the Mozambican context—where women tend to want fewer children than men [11]—it would be important to further explore how the planning process takes place, how and which goals are set, and what steps are required to achieve those goals. The role of family planning should be examined, as some Mozambican women reported that learning about and accessing family planning was the turning point for starting to envision their lives differently, because they could plan and control their reproductive lives.

Other elements identified by women—such as independence, being free and having an active participation in life—have been accounted for in the literature about women’s empowerment [18, 33]. Although these elements do not directly confer empowerment, they have an enabling role as essential pre-conditions or resources for empowerment and the exercise of agency [8]. As women in this study described, financial independence increases their negotiation capacity within the household but this may not lead to agency, i.e. to enact their reproductive decisions [5]. This is aligned with studies reporting on the limited impact of programs that aim to improve sexual and reproductive outcomes for women and girls by improving access to financial assets in low- and middle-income countries such as Mozambique [39]. The chances of success of such programs are increased when approaches that support women to act on their decisions are also in place, such as building consciousness and knowledge about sexual and reproductive health and rights, and self-efficacy practices [39].

### Strengths and limitations

This study puts women at the center of empowerment, giving them a voice and expanding on the conceptualization and operationalization of women’s empowerment, focusing on sexual and reproductive health. However, some limitations need to be considered. The study applied a convenience sampling to recruit participants, both at the health facility and at the community levels which could have led to bias in the selection of participants. Women who attended the health center or were known to traditional leaders could be more familiar with contraception methods and more aware of their choices/ more empowered than those not identified during the recruitment process. In addition, the interviewer, being an outsider, could have also introduced bias, leading to socially desirable answers from the participants. However, this could have been minimized by the presence of the local research assistant.

## Conclusions

Through women's views, meanings and lived experiences, this study identified key elements of the empowerment process in the Mozambican context. Women with empowerment were perceived by participants as independent, active participants in decision-making, and free. These characteristics have been recognized in the literature as key enablers for women’s empowerment process. Crucial elements of how women exert power for reproductive choices were also identified, namely: the ability to plan the number and timing of pregnancies (facilitated by accessing family planning services and information), negotiation capacity with the partner (by voicing choices and influencing decision), or sole decision-making based on a sense of entitlement. These elements provide insights into expanding current methods of conceptualizing, operationalizing and measuring women’s sexual and reproductive empowerment. This study recommends that further research is performed around the identified elements, exploring and testing their ability to capture and describe empowerment. Furthermore, this study highlights the importance of including women’s views and perceptions for advancements in research that ultimately can be more meaningful for women’s lives and realities.

### Supplementary Information

**Additional file 1. ** Interview guide. List of questions used to interview women in Mozambique.

**Additional file 2: Table S1.** Key characteristics of the participants from urban and rural areas of Maputo (including Maputo city) and Nampula provinces. Provides an overview of key sociodemographic characteristics, fertility and family planning practices of all participants

## Data Availability

Data supporting results in this article is filed and safely locked away. The corresponding author is ready to avail the said data on reasonable request.

## Abbreviations

SDGs: Sustainable development goals
DPS: Provincial Health Directorate (Direcção Provincial de Saúde)

## References

[1] United Nations (UN). SDG indicator metadata (SDG 5). Department of Economic and Social Affairs Statistics; 2022. https://unstats.un.org/sdgs/metadata/files/Metadata-05-06-01.pdf.

[2] Upadhyay UD, Gipson JD, Withers M, Lewis S, Fraser CS, Huchko MJ (2014). Women’s empowerment and fertility: a review of the literature. Soc Sci Med.

[3] Prata N, Fraser A, Huchko MJ, Gipson JD, Withers M, Lewis S (2017). Women’s empowerment and family planning: a review of the literature. J Biosoc Sci..

[4] Richardson RA (2017). Measuring women’s empowerment: a critical review of current practices and recommendations for researchers. Soc Indic Res.

[5] Edmeades J, Mejia C, Parsons J, Sebany M. Conceptual framework for reproductive empowerment: empowering individuals and couples to improve their health (Background Paper). 2018. https://www.icrw.org/wp-content/uploads/2018/10/Reproductive-Empowerment-Background-Paper_100318-FINAL.pdf.

[6] Pratley P, Sandberg JF (2017). Refining the conceptualization and measurement of women’s empowerment in sub-saharan Africa using data from the 2013 Nigerian demographic and health survey. Soc Indic Res.

[7] Ewerling F, Lynch JW, Victora CG, van Eerdewijk A, Tyszler M, Barros AJD (2017). The SWPER index for women’s empowerment in Africa: development and validation of an index based on survey data. Lancet Glob Health.

[8] Kabeer N (1999). Resources, agency, achievements: reflections on the measurement of women’s empowerment. Dev Change..

[9] Capurchande R, Coene G, Roelens K, Meulemans H (2017). “If I have only two children and they die… who will take care of me?”—A qualitative study exploring knowledge, attitudes and practices about family planning among Mozambican female and male adults. BMC Womens Health..

[10] Ministério do Género Criança e Acção Social (MGCAS). Perfil de género de Moçambique. Maputo, Moçambique; 2016. file:///C:/Users/fioca/Downloads/Perfil de Género de Moçambique.pdf.

[11] Ministério da Saúde (MISAU), Instituto Nacional de Estatística (INE), ICF. Inquérito de indicadores de imunização, malária e HIV/SIDA em Moçambique 2015. Maputo, Moçambique. Rockville, Maryland, EUA; 2015. https://dhsprogram.com/pubs/pdf/AIS12/AIS12.pdf.

[12] United States Agency for International Development (USAID). Improved family planning initiative. 2022. https://www.usaid.gov/mozambique/fact-sheet/improved-family-planning-initiative-usaid-ifpi.

[13] Countdown to 2030. Countdown 2030 country profile: Mozambique. 2023. https://data.unicef.org/countdown-2030/country/Mozambique/1/. Accessed 4 Jul 2023.

[14] Instituto Nacional de Estatística. A situação socioeconómica da juventude em Moçambique.. Maputo, Mozambique; 2023. https://mozambique.unfpa.org/sites/default/files/pub-pdf/condicoes_socioeconomicas_da_juventude_-_mario-22-06-23_0_0.pdf.

[15] Gebregziabher FH, Pijuan Sala A, Massarongo Chivulele FAP, Massingue ACA, Casal J, De Lemos Botelho Barreto RJ, et al. Mozambique economic update: setting the stage for recovery. World Bank. Washington D.C; 2021. https://documents.worldbank.org/en/publication/documents-reports/documentdetail/931171614625070870/mozambique-economic-update-setting-the-stage-for-recovery.

[16] United Nations Programme on HIV/AIDS (UNAIDS). HIV and AIDS estimates. Country factsheet. Mozambique 2022. 2022. https://www.unaids.org/en/regionscountries/countries/mozambique. Accessed 6 Jan 2024.

[17] Ministério da Saúde (MISAU). Plano estratégico do séctor saúde 2014–2019 (2020–2024). Maputo, Mozambique; 2014. https://www.misau.gov.mz/index.php/planos-estrategicos.

[18] Kabeer N. Reflection on the measurement of women empowerment. In: Anne Sisask, editor. Discuss women’s empower. SIDA; 2001. p. 136. https://cdn.sida.se/publications/files/sida984en-discussing-womens-empowerment---theory-and-practice.pdf.

[19] Malhotra A, Schuler SR, Boender C. Conceptualizing and measuring women’s empowerment as a variable in international development. Pap Prep World Bank Work Poverty Gend New Perspect. 2003;9. https://www.ssatp.org/sites/ssatp/files/publications/HTML/Gender-RG/Sourcedocuments/Technical Reports/GenderResearch/TEGEN5MeasuringWomen%27sEmpowermentICRW2002.pdf. Accessed 1 Apr 2023.

[20] Do M, Kurimoto N (2012). Women’s empowerment and choice of contraceptive methods in selected African countries. Int Perspect Sex Reprod Health..

[21] Karp C, Wood SN, Galadanci H, Sebina Kibira SP, Makumbi F, Omoluabi E (2020). ‘I am the master key that opens and locks’: presentation and application of a conceptual framework for women’s and girls’ empowerment in reproductive health. Soc Sci Med.

[22] Family Planning 2020. Family planning 2020: rights and empowerment principles for family planning. 2019. https://www.fp2030.org/resources/resources-family-planning-2020-rights-empowerment-principles-family-planning/.

[23] Castro Lopes S, Constant D, Fraga S, Bique Osman N, Correia D, Harries J (2021). Socio-economic, demographic, and behavioural determinants of women’s empowerment in Mozambique. PLoS ONE.

[24] Castro Lopes S, Constant D, Fraga S, Harries J (2022). How women’s empowerment influences fertility-related outcomes and contraceptive practices: a cross-sectional study in Mozambique. PLOS Glob Public Health..

[25] Castro Lopes S, Constant D, Fraga S, Osman NB, Harries J (2022). “There Are Things We Can Do and There Are Things We Cannot Do.” A Qualitative Study About Women’s Perceptions on Empowerment in Relation to Fertility Intentions and Family Planning Practices in Mozambique. Front Glob Women’s Health..

[26] Sen A, Nussbaum S (1993). Capability and well-being. Qual life.

[27] Sen A. Development as freedom. Oxford University Press; 1999. https://books.google.co.za/books?id=NQs75PEa618C.

[28] Cornwall A. Addressing the preconditions: women’s rights and development. 2002; http://www.pathwaysofempowerment.org/archive_resources/addressing-the-preconditions-women-s-rights-and-development-in-financing-gender-equality-commonwealth-perspectives.

[29] Hinson L, Edmeades J, Murithi L, Puri M (2019). Developing and testing measures of reproductive decision-making agency in Nepal. SSM Popul Health..

[30] Bagnoli A (2009). Beyond the standard interview: the use of graphic elicitation and arts-based methods. Qual Res.

[31] NVivo qualitative data analysis software; Version 12. QSR International Pty Ltd.; 2020.

[32] Braun V, Clarke V. Thematic analysis. APA Handb Res methods Psychol. 2012. p. 57–71.

[33] Eerdewijk A van, Wong F, Vaast C, Newton J, Tyszler M, Pennington A, et al. White paper: a conceptual model on women and girls’ empowerment. Amesterdam; 2017. https://www.kit.nl/publication/white-paper-conceptual-model-of-women-and-girls-empowerment/.

[34] Donald A, Koolwal G, Annan J, Falb K, Goldstein M. Measuring women’s agency. Policy Res. Work. Pap. World Bank, Washington, DC; 2017 Jul. https://openknowledge.worldbank.org/handle/10986/27955.

[35] The Bill and Melinda Gates Foundation (BMGF). Gender equality toolbox. What gets measured matters. A methods note for measuring women and girls empowerment. 2019. https://www.gatesgenderequalitytoolbox.org/wp-content/uploads/BMGF_Methods-Note-Measuring-Empowerment-1.pdf.

[36] Peterman A, Schwab B, Roy S, Hidrobo M, Gilligan DO (2021). Measuring women’s decision making: indicator choice and survey design experiments from cash and food transfer evaluations in Ecuador, Uganda and Yemen. World Dev.

[37] Kibira SPS, Karp C, Wood SN, Desta S, Galadanci H, Makumbi FE (2020). Covert use of contraception in three sub-Saharan African countries: a qualitative exploration of motivations and challenges. BMC Public Health.

[38] International Food Policy Research Institute (IFPRI). Women’s empowerment in agriculture index. Guides and instruments. 2023. 2023. https://weai.ifpri.info/weai-resource-center/guides-and-instruments/. Accessed 16 Jun 2023.

[39] Zelalem D, Worku A, Alemayehu T, Dessie Y. Association of effective spousal family planning communication with couples’ modern contraceptive use in Harar, Eastern Ethiopia. Open Access J Contracept. New Zealand; 2021; 12:45–62. https://www.dovepress.com/association-of-effective-spousal-family-planning-communication-with-co-peer-reviewed-article-OAJC. 10.2147/OAJC.S285358 PMC7924133 33679142

