# Supplementary material for: Women's voices and meanings of empowerment for reproductive decisions: a qualitative study in Mozambique
Source: Reprod Health. 2024 Feb 2;21:16. doi: 10.1186/s12978-024-01748-7 (PMC10837942; doi:10.1186/s12978-024-01748-7)
Supplement: Supplementary file 1 — Additional file 1. Interview guide. List of questions used to interview women in Mozambique. [file 12978_2024_1748_MOESM1_ESM.pdf]

INTERVIEW GUIDE

INTRODUCTION

Thank you for agreeing to participate in this study. As explained before, we are going to have a conversation about family planning and becoming a mother, specifically we want to hear your opinions about these issues. Please remember that there are no right or wrong answers and I just want to hear your opinions. I will audio-record our conversation so I can remember everything you said later. However, this recording will be deleted after transcribing our conversation. Do you have any questions? Do you give your permission for us to proceed?

Let us start then! I am going to put the recorder on now.

SOCIO-DEMOGRAPHIC INFORMATION

Province: ____ **Participants study ID**:|________|

Name:____ **Age:** |___|___|

Marital status: single |___| married or in union |___| divorced/separated|___| widow |___|

If married, does your husband lives with you or if he working abroad:__________________

Current employment situation: employed |___| unemployed |___|

Profession/Occupation:_____________________________

Number of pregnancies: |___|___| **Parity:** |___|___| **Number of live children:** |___|___|

Ethnicity: ______________ **Size of the household**: |___|___|

Current use of contraception: Yes |___| No |___|

if yes, indicate which: _____________________________________

Ever use of contraception: Yes | | No | |

Health centre/community of recruitment: _______________________________

Place of residence (neighbourhood, district):____

Date of recruitment:|___|___|/|___|___|/|___|___|

Date of interview: |___|___|/|___|___|/|___|___| Start time:____:_____ End time:____:_____

Version 5
12 Feb 2020

QUESTIONS

Overarching question (guiding question): Exploring the decisions about fertility and family planning over a timeline

I would like us to focus in your life and in your experiences. Let’s think about important moments that were part of your life, for example, the day of your first menstruation, your wedding, your first pregnancy, delivery, following pregnancies, use of contraception, your first paid job (choose examples adjusted to the participant), among other moment. Once we identified these important events in your life, I would like you to tell me about each of these moments.

Moments to consider:

- Menstruation

Try to remember when you had your menstruation for the first time. How old were you? What do you remember from that moment?

Explore other memories – what did you feel? What changed? Was she studying? What happened next (rituals?)

- Wedding

What do you think about marriage? How old were you when you got married? What made you decide to get married? What changed in your life?

Explore: forced vs. decision/choose to be married; need to be an official/traditional marriage – lobolo; Was it important for her status? What changed? What were the expectations of others and herself?

- Pregnancy/maternity/contraception

I. Decision-making about fertility/reproduction and family planning WITHIN the family

Now let us consider specifically the decisions about woman falling/not falling pregnant and becoming a mother:

1. Until when did you use contraception? And after getting married, until getting pregnant?

2. How was the moment when you decided having/not having a child (for those who do not have children)?

Prompts: what is important for you? Who was involved in that decision? What changed?

3. What are the plans for the future, would you like to have children? What are the reasons for wanting/not wanting to have children? (choose the right option)

   Prompts: What influenced this decision/choice? Can you look for contraception by yourself?

4. In these moments of decision-making in your house, what is the role of the man/your partner?

Prompts: Do you have the option/can you discuss these issues with your husband or other person who makes or participates in the decision-making? Explore relational and negotiation aspects.

5. How women in your community/society make decisions about family planning, having children, etc? Think about what can hinder women of making decisions alone or on the other hand, can't help women make decisions alone.

II. Decision-making about fertility and family planning within the CONTEXT of the health services

1. What do you think about the health workers who did the follow up of your pregnancy/family planning? How were they important for the decisions we just spoke about? Can you please give some examples?

2. Overall, what is the role of the health providers in the decisions of women about the number of children to have or the use of contraception? Can you please give some examples?

Prompts: Inform women? Involve partners? In which ways this can help women having more power to choose freely or decide on what she think it is best?

Other moments:

- Employment

Depending on what her occupation is: Do you have access to income? How do you manage your money at home? (Income management? Decisions about spending?)

- Death of a close family member

Situation that could have increased the woman vulnerability and to decisions about of school dropping, early marriage, etc.

- Violence exposure (explore only if referred by the woman)

In relation to her decision making – if exists or existed, is it inside the household? Is it her partner? In which situations? How does she react or acts on it?

- Participation in a community group: association, credit group

Income that she can manage and keep her business? Access to resources? Land, information?

<u>Looking at the women and men in the community/society:</u>

DECISION-MAKING PROCESS/CHOICE AND MEANING OF POWER

1. **What do you think about the Mozambican woman? Do you think that the women of today are different from the women from before (some time ago)? Were there changes or everything is still the same? Tell me your thoughts about his.**

Version 5
12 Feb 2020

2. **What are the roles/responsibilities that are from men and what are the roles/responsibilities of women within the household and in the community/society in Mozambique?**

Prompts: tasks distribution/division based on sex, behaviours determined by the family relationships and hierarchies.

3. **Within the roles you described, in which situations men and women can have the same roles/responsibilities or they can swop, for example? What does it mean?**

Prompts: is there lose of powers? Of social status? Is there transfer of roles and powers? What are the barriers and consequences?

4. **Why do you think there are these differences between men and woman? What does it mean?**

Prompts: Different power – meaning and perceptions about the concept of power – being capable of decision-making, having control, having the capacity, the strength, strengthening, freedom, do what she considers the best.

5. **What do you think about the Mozambican women who makes decisions alone about their lives? When does it happen?**

Prompts: Is it possible for a woman to do it? Why or why not? What are the consequences and what levels? Is there discrimination or punishment in the community?

6. **What do you think can contribute to the Mozambican woman to have more power (decision/choice) in your community/society?**

7. **In which ways the community/society organization can influence the power of a woman and men to decide or make choices?**

Prompts: role of the traditional leaders? Rituals? Women's groups or associations?

This was our last question. Is there anything that you would like to add?

Thank you for your time.

**Observações/comentários:**
