# Supplementary material for: Women's voices and meanings of empowerment for reproductive decisions: a qualitative study in Mozambique
Source: Reprod Health. 2024 Feb 2;21:16. doi: 10.1186/s12978-024-01748-7 (PMC10837942; doi:10.1186/s12978-024-01748-7)
Supplement: Supplementary file 2 — Additional file 2: Table S1. Key characteristics of the participants from urban and rural areas of Maputo (including Maputo city) and Nampula provinces. Provides an overview of key sociodemographic characteristics, fertility and family planning practices of all participants [file 12978_2024_1748_MOESM2_ESM.docx]

**Additional material**

Table 1: Key characteristics of the participants from urban and rural areas of Maputo (including Maputo city) and Nampula provinces.

| **#** | **Region** | **Local** | **Site** | **Age**  **(years)** | **Civil status** | **Education (years)** | **Employed** | **Age of 1st pregnancy** | **Number of pregnancies** | **Type of contraception currently used** | **Ever use of contraception** | **Type of contraception previously used** |
| --- | --- | --- | --- | --- | --- | --- | --- | --- | --- | --- | --- | --- |
| 1 | M | Urban | HC | 32 | Married | 15 | yes | 26 | 2 | Pill | yes | Pill |
| 2 | M | Urban | HC | 34 | Married | 16 | no | 24 | 3 | Pill | yes | Pill and condom |
| 3 | M | Urban | HC | 24 | single | 10 | yes | 20 | 1 | Pill | yes | Implant |
| 4 | M | Urban | HC | 25 | Married | 7 | no | 17 | 2 | Injection | yes | Injection |
| 5 | M | Urban | HC | 34 | Married | 17 | yes | 27 | 2 | Pill | yes | Pill |
| 6 | M | Urban | HC | 25 | Single | 16 | yes | na | 0 | Pill and condom | yes | Condom |
| 7 | M | Urban | HC | 42 | Married | 9 | no | 23 | 1 | Injection | yes | Pill |
| 8 | M | Urban | C | 29 | Single | 16 | yes | na | 0 | Condom | yes | Pill |
| 9 | M | Urban | C | 37 | Single | 12 | yes | * | 1 | Condom | yes | Pill |
| 10 | M | Urban | C | 25 | Single | 12 | yes | na | 0 | Condom | yes | Vaginal ring |
| 11 | M | Urban | C | 39 | Single | 18 | yes | 19 | 1 | No use | yes | Pill |
| 12 | M | Urban | C | 42 | Union | 8 | yes | 19 | 5 | Implant and condom | yes | Injection |
| 13 | M | Urban | C | 40 | Union | 10 | yes | 15 | 4 | Condom (sometimes) | yes | Injection |
| 14 | M | Urban | C | 34 | Divorced | 12 | yes | 24 | 2 | IUD | yes | Pill |
| 15 | M | Rural | HC | 33 | Divorced | 10 | no | 21 | 3 | Condom | yes | Injection and pill |
| 16 | M | Rural | HC | 21 | Union | 6 | no | 15 | 2 | Injection | yes | Injection and pill |
| 17 | M | Rural | HC | 43 | Married | 5 | no | 15 | 6 | Condom | yes | Injection and pill |
| 18 | M | Rural | HC | 26 | Union | 7 | no | 17 | 1 | No use | yes | Condom |
| 19 | M | Rural | HC | 31 | Union | 0 | yes | 16 | 3 | Implant | yes | Pill and condom |
| 20 | M | Rural | HC | 18 | Single | 10 | no | 17 | 1 | Injection and condom | yes | Condom |
| 21 | M | Rural | HC | 23 | Married | 11 | no | 15 | 5 | Pill | yes | Pill |
| 22 | M | Urban | HC | 20 | Union | 10 | yes | 18 | 1 | Injection | yes | Injection and condom |
| 23 | M | Urban | HC | 44 | Married | 8 | no | 16 | 4 | Implant | yes | Pill |
| 24 | M | Urban | HC | 32 | Union | 12 | yes | 25 | 3 | Pill | yes | Pill |
| 25 | M | Urban | HC | 32 | Divorced | 0 | no | 15 | 4 | Pill | yes | Injection and condom |
| 26 | M | Urban | HC | 33 | Married | 12 | yes | 18 | 4 | IUD | yes | Implant and pill |
| 27 | M | Urban | HC | 30 | Union | 10 | yes | 17 | 3 | Pill | yes | Injection |
| 28 | M | Urban | HC | 22 | Single | 9 | no | 18 | 1 | Injection | yes | Implant |
| 29 | M | Urban | C | 42 | Divorced | 12 | yes | 17 | 4 | Condom | no |  |
| 30 | M | Urban | C | 39 | Married | 6 | yes | 17 | 4 | Injection | yes | Pill |
| 31 | M | Urban | C | 47 | Married | 5 | no | 18 | 5 | No use | yes | Injection |
| 32 | M | Urban | C | 37 | Single | 7 | no | 21 | 5 | Injection and condom | yes | IUD |
| 33 | M | Urban | C | 48 | Widow | 7 | no | 20 | 6 | No use | yes | Injection and condom |
| 34 | M | Urban | C | 20 | Single | 10 | no | 16 | 1 | No use | yes | Injection and condom |
| 35 | M | Rural | C | 39 | Union | 0 | no | 17 | 7 | No use | yes | Injection |
| 36 | M | Rural | C | 33 | Divorced | 4 | no | 19 | 7 | No use | yes | Pill and implant |
| 37 | M | Rural | C | 25 | Union | 6 | no | 16 | 3 | Condom | yes | Injection and pill |
| 38 | M | Rural | C | 31 | Union | 6 | no | 16 | 3 | Sterilization | yes | Injection |
| 39 | M | Rural | C | 36 | Union | 8 | no | 14 | 5 | Injection | yes | Pill and implant |
| 40 | N | Urban | HC | 30 | Union | 4 | no | 16 | 6 | Injection | no |  |
| 41 | N | Urban | HC | 36 | Union | 10 | no | 18 | 5 | Injection | yes | Injection |
| 42 | N | Urban | HC | 19 | Union | 11 | no | 19 | 1 | Injection | no |  |
| 43 | N | Urban | HC | 21 | Union | 12 | no | 18 | 1 | Injection | yes | Condom |
| 44 | N | Urban | HC | 23 | Divorced | 12 | yes | 20 | 3 | Injection | no |  |
| 45 | N | Urban | HC | 20 | Single | 12 | yes | na | 0 | Implant | no |  |
| 46 | N | Urban | HC | 40 | Union | 12 | yes | 19 | 6 | Injection | yes | Condom |
| 47 | N | Urban | HC | 18 | Union | 7 | no | 18 | 1 | No use | yes | Condom |
| 48 | N | Urban | HC | 25 | Union | 10 | no | 20 | 3 | Injection | Yes | Implant |
| 49 | N | Urban | HC | 21 | Union | 12 | no | 17 | 2 | Pill | yes | Injection |
| 50 | N | Urban | C | 32 | Union | 10 | no | 17 | 4 | Injection | yes | Condom |
| 51 | N | Urban | C | 46 | Widow | 10 | no | * | 15 | No use | Yes | Injection and pill |
| 52 | N | Urban | C | 47 | Union | 10 | no | 20 | 8 | No use | no |  |
| 53 | N | Urban | C | 35 | Union | 9 | no | 19 | 5 | Injection | yes | Injection |
| 54 | N | Rural | HC | 21 | Single | 9 | no | * | 3 | No use | no |  |
| 55 | N | Rural | HC | 23 | Union | 8 | no | 20 | 2 | No use | yes | Injection |
| 56 | N | Rural | HC | 36 | Union | 0 | no | 18 | 7 | Injection | no |  |
| 57 | N | Rural | HC | 27 | Union | 8 | no | 21 | 5 | Pill | yes | Pill |
| 58 | N | Rural | HC | 18 | Union | 8 | no | 17 | 1 | No use | no |  |
| 59 | N | Rural | HC | 22 | Union | 5 | no | 20 | 2 | Injection | yes | Injection |
| 60 | N | Rural | HC | 19 | Union | 4 | no | 18 | 1 | No use | no |  |
| 61 | N | Rural | HC | 22 | Union | 4 | no | 18 | 4 | No use | no |  |
| 62 | N | Rural | HC | 20 | Single | 0 | no | 19 | 1 | Injection | no |  |
| 63 | N | Rural | C | 44 | Union | 8 | no | 19 | 9 | Injection | yes | Injection |
| 64 | N | Rural | C | 30 | Union | 4 | no | 18 | 12 | No use | yes | Injection, Pill and Condom |

# - interview number; Region: M – Maputo, N-Nampula; Site: HC – Health center, C- Community; IUD – Intrauterine device; *missing
